# Carbon and nitrogen metabolic regulation in freshwater plant *Ottelia alismoides* in response to carbon limitation: A metabolite perspective

**DOI:** 10.3389/fpls.2022.962622

**Published:** 2022-09-15

**Authors:** Wenmin Huang, Shijuan Han, Liyuan Wang, Wei Li

**Affiliations:** ^1^Aquatic Plant Research Center, Wuhan Botanical Garden, Chinese Academy of Sciences, Wuhan, China; ^2^University of Chinese Academy of Sciences, Beijing, China; ^3^Research Center for Ecology, College of Science, Tibet University, Lhasa, Tibet, China

**Keywords:** carbon metabolism, glycolysis, low CO_2_ stress, metabolic profiling, N metabolism, TCA cycle

## Abstract

Carbon and nitrogen metabolism are basic, but pivotal metabolic pathways in plants and are tightly coupled. Maintaining the balance of carbon and nitrogen metabolism is critical for plant survival. Comprehensively revealing the metabolic balance of carbon–nitrogen interactions is important and helpful for understanding the adaptation of freshwater plants to CO_2_ limited aqueous environment. A comprehensive metabolomics analysis combined with physiological measurement was performed in the freshwater plant *Ottelia alismoides* acclimated to high and low CO_2_, respectively, for a better understanding of how the carbon and nitrogen metabolic adjustment in freshwater plants respond to carbon limitation. The present results showed that low CO_2_ acclimated *O. alismoides* exhibited significant diurnal titratable acidity and malate fluctuations, as well as an opposite diel pattern of starch change and high enzymatic activities required for crassulacean acid metabolism (CAM) photosynthesis, which indicates that CAM was induced under low CO_2_. Moreover, the metabolomic analysis showed that most intermediates of glycolysis, pentose phosphate pathway (PPP) and tricarboxylic acid (TCA) cycle, were increased under low CO_2_, indicative of active respiration in low-CO_2_-treated *O. alismoides*. Meanwhile, the majority of amino acids involved in pathways of glutamate and arginine metabolism, aspartate metabolism, and the branched-chain amino acids (BCAAs) metabolism were significantly increased under low CO_2_. Notably, γ-aminobutyric acid (GABA) level was significantly higher in low CO_2_ conditions, indicating a typical response with GABA shunt compensated for energy deprivation at low CO_2_. Taken together, we conclude that in low-CO_2_-stressed *O. alismoides*, CAM photosynthesis was induced, leading to higher carbon and nitrogen as well as energy requirements. Correspondingly, the respiration was greatly fueled *via* numerous starch degradation to ensure CO_2_ fixation in dark, while accompanied by linked promoted N metabolism, presumably to produce energy and alternative carbon sources and nitrogenous substances for supporting the operation of CAM and enhancing tolerance for carbon limitation. This study not only helps to elucidate the regulating interaction between C and N metabolism to adapt to different CO_2_ but also provides novel insights into the effects of CO_2_ variation on the metabolic profiling of *O. alismoides*.

## Introduction

Carbon and nitrogen are the two most abundant nutrient elements in plants, and their metabolisms are the two most basic but pivotal metabolic pathways in plants, and they are tightly coupled (Zhang et al., 2018). Both these two important metabolic processes require carbon skeletons, reducing power, and energy supplied from photosynthetic electron transport or respiration (Sweetlove et al., 2010). The carbon source and energy required for nitrogen metabolism could be provided by carbon metabolism, while nitrogen metabolism could provide photosynthetic pigments and enzymes for carbon metabolism (Nunes-Nesi et al., 2010). Specific to amino acid synthesis, the C skeletons for amino acid synthetic pathways are produced in different sectors of the respiratory pathways. The majority of the photosynthetic fixed C is converted into phosphoenolpyruvate (PEP) *via* glycolysis and further invested in the synthesis of organic acids through the tricarboxylic acid (TCA) cycle, such as into 2-oxoglutarate and oxaloacetate, which are the main organic acids used for amino acid synthesis (Huppe and Turpin, 1994).

The carbon and nitrogen metabolism involves extensive regulation between the two pathways in plants (Huppe and Turpin, 1994). The active adjustment of carbon and nitrogen metabolism would affect the production and conversion of the photosynthetic products, as well as the synthesis of proteins and absorption of other nutrients. Thus, maintaining the balance of carbon and nitrogen metabolism is critical for plant survival (Krapp and Traong, 2005; Nunes-Nesi et al., 2010). Previous studies have revealed that carbon and nitrogen metabolism in photoautotrophs must closely interact and coordinate when adapting to variable environmental conditions. The treatments that inhibit photosynthetic carbon fixation, such as CO_2_ deprivation and photosynthetic inhibitors in algae, limit the supply of carbon skeletons and restrict the assimilation of nitrogen into amino acids (Romero et al., 1985). In low CO_2_ stressed maize seedlings, the assimilation of N was regulated by the capacity of photosynthesis and the availability of stored carbohydrates (Pace et al., 1990). The review of higher terrestrial plants has shown that elevated CO_2_ improved nitrogen use efficiency and promoted dark respiration, especially in soybean (Leakey et al., 2009).

Macrophytes are important primary producers in productive freshwater ecosystems. The growth of macrophytes is frequently limited by the availability of carbon, as the low rates of CO_2_ diffusion in the aqueous environment (∼10,000 times lower than in air) and the external boundary layer formed around organisms constrain the uptake of inorganic carbon (Maberly and Madsen, 1998; Iversen et al., 2019). Comprehensively revealing the regulation of carbon and nitrogen metabolism, as well as their interactions are important and helpful for understanding the adaptation of freshwater plants to lower CO_2_ stress. However, the studies on the photosynthetic carbon and nitrogen metabolism response to CO_2_ limitation in freshwater plants are fragmented and limited, with the research emphasis frequently being focused on induction or switching of the photosynthetic pathways, as well as the small group of compounds’ metabolism from the induced photosynthetic pathway, such as the crassulacean acid metabolism (CAM) under limited CO_2_ conditions (Baattrup-Pedersen and Madsen, 1999; Klavsen and Maberly, 2010). CAM could serve to conserve water by minimizing gaseous exchange during the day for terrestrial plants, however, it is also a carbon-conserving mechanism by reducing respiratory carbon loss (Silvera et al., 2010). Recently, it has been shown that *O. alismoides* (a member of the Hydrocharitaceae) can operate CAM facultatively at low CO_2_, and at night, the activated phosphoenolpyruvate carboxylase (PEPC) fixes CO_2_, causing nocturnal malic acid accumulation and concomitant depletion of starch. During the day, this C4 acid is decarboxylated to produce CO_2_ that is captured by ribulose-bisphosphate carboxylase/oxygenase (Rubisco) into the Calvin–Benson cycle (Shao et al., 2017). Although such studies are essential and pivotal, they could not reveal the coordinating networks of carbon and nitrogen metabolic responses to low CO_2_ stress. Nevertheless, large metabolite datasets are essential to parameterize flux balance models (Cheung et al., 2014; Shameer et al., 2018), which could be useful for estimating the interaction between C and N metabolism.

*Ottelia alismoides* generally inhabits shallow waters and forms dense biomass, resulting in large diel fluctuations and low concentrations of CO_2_ (Shao et al., 2017). To better understand the carbon and nitrogen metabolic adjustment, as well as the carbon–nitrogen interactions of freshwater plant *O. alismoides* in response to carbon limitation, integrated measurements of diel change of photosynthates, soluble proteins, free amino acids, and photosynthetic enzyme activity, and a comprehensive metabolomics analysis were performed in *O. alismoides* plants acclimated to high and low CO_2_, respectively. This work will provide deeper and more reliable insights into the response of *O. alismoides* to different levels of CO_2_.

## Materials and methods

### Plant material

In April 2020, *O. alismoides* seeds were germinated on sterilized soil covered with sterile tap water (alkalinity ∼2.0 mequiv L^–1^, TP 1.61 μmol L^–1^, TN 0.1 mmol L^–1^) in three plastic pots. They were put in a growth chamber set at 28°C with a 14/10 h photoperiod (120 μmol photons m^–2^ s^–1^). About a month and a half later, every two seedlings (∼10 cm height) were transplanted into a plant pot (15 cm diameter and 10 cm height), and a total of eighteen pots were placed in a tank (64 cm high) located on the flat roof of the laboratory for further expanded cultivation. The seedlings in the tank received natural light and were fully submerged during the whole cultivation period.

### Acclimation to different CO_2_

After ∼8 weeks of growth in the tank, sixteen pots of *O. alismoides* with similar height (∼30 cm) and lots of oval mature leaves were transferred into eight plastic buckets (25 × 25 × 35 cm), two pots per bucket, used for different CO_2_ treatments. All the eight buckets were placed in a growth room at 25 ± 2°C and illuminated by white fluorescence tubes with a 14/10 h photoperiod (08:00–22:00 light, ∼130 μmol photons m^–2^ s^–1^). Four buckets are for the high CO_2_ treatment and the other four are for the low CO_2_ treatment. The *O. alismoides* plants were cultured with tap water and treated with high and low CO_2_, respectively. In high CO_2_ treatment (HC), CO_2_-saturated tap water was added to the buckets two times each day to produce HC treatment, maintaining the pH at 6.7–7.0. The resultant CO_2_ over the whole experimental period was between 457 and 864 μmol L^–1^, with a mean of 649 μmol L^–1^. In low CO_2_ treatment (LC), low CO_2_ was produced by natural photosynthesis of the experimental plants, which consumed the inorganic carbon, and the pH was increased from 8.1 to 10.0, generating a CO_2_ range of ∼0.03–11.5 μmol L^–1^ and a mean CO_2_ of 1.5 μmol L^–1^ during the experimental period. CO_2_ concentration was calculated from temperature and pH, and alkalinity was measured by Gran titration based on the equations reported by Maberly (1996). The conditions in low and high CO_2_ treatments are presented in Supplementary Table 1. After ∼20 days of treatment with different CO_2_, the newly produced oval mature leaves were sampled at 21:30 (at the end of the light period) and 07:30 (toward the end of the dark period) from different *O. alismoides* plants from different buckets, respectively, for physiological measurement and metabolomics analyses. Leaf samples per treatment were taken in triplicate from three different plants in different buckets, and samples at both times were collected from the same plant. After collection, all the leaf samples were stored immediately at **−**80°C before measurement.

### Measurement of titratable acidity and malic acid

The titratable acidity was measured following the previous method with minor modifications (Zhang et al., 2014). A total of 12 ml CO_2_-free water was added to the known fresh weight of (FW) leaf samples (∼0.2 g) in 15 ml tightly sealed screw-cap plastic tubes and were incubated in a boiling water bath for 60 min. After cooling, the acidity of the samples was measured by titration with 0.01 N NaOH to a pH endpoint of 8.3. The malic acid content was detected as described previously (Han et al., 2020). The leaf samples were extracted with pre-chilled 5% (v/v) perchloric acid and then centrifuged. The pH of the resultant supernatants was adjusted to 3.0–3.5 with saturated K_2_CO_3_ solution. After that, the supernatants were centrifuged again and filtered through 0.22 μm filters, and then these solutions were ready for measurement with high-performance liquid chromatography (HPLC). The malic acid concentration was quantitatively determined by analyzing the chromatographic data.

### Measurement of starch

Starch content was determined according to Smith and Zeeman (2006) and Shao et al. (2017). A total of 5 ml of 80% ethanol was added to the frozen leaves (∼0.3 g FW) for homogenization. Then, the homogenate was incubated in a boiling water bath for 5 min and centrifuged at 6,000 × *g* for 10 min at room temperature. The supernatant was discarded while the pellet was kept for the next step. This ethanol extraction was repeated two times, and then pure water was added to the ethanol-extracted pellets. After a thorough homogenization, the homogenate was incubated in a boiling water bath for 10 min. After the homogenate was cooled to room temperature, 0.2 M Na acetate (pH 5.5), α-amyloglucosidase, and α-amylase were added to the homogenate, and the reaction mixture was incubated at 37°C for 4 h. Then, it was ready for glucose measurements through the amyloglucosidase assay (Smith and Zeeman, 2006).

### Measurement of soluble carbohydrates, soluble proteins, and free amino acids

The content of soluble carbohydrates was determined by the phenol/sulfuric acid method and expressed as mg g^–1^ FW (Taylor, 1995; Jain et al., 2017). The extraction and assay of soluble proteins were based on the methods described by Bradford (1976) with the bovine serum albumin for calibration. The free amino acids were determined according to the ninhydrin method (Yokoyama and Hiramatsu, 2003; Xian et al., 2020).

### Measurement of photosynthetic enzyme activity

As the key photosynthetic enzymes function in CAM metabolism, the activities of Rubisco, PEPC, and pyruvate phosphate dikinase (PPDK regenerates PEP to provide a substrate for PEPC) need to be determined. The extraction and measurement of Rubisco, PEPC, and PPDK were performed according to the methods described by Zhang et al. (2014) and Shao et al. (2017). Enzyme activities were calculated from the rates of NADH variation at 340 nm.

### Metabolic profiling of *Ottelia alismoides* by HPLC-MS/MS

The low molecular weight metabolites of *O. alismoides* were extracted and determined by quasi-targeted metabolomics according to the method reported in Want et al. (2012). Leaves (∼0.1 g FW) were grounded with pre-chilled 80% methanol, and then the homogenates were incubated on ice for 5 min. After that, the samples were centrifuged at 15,000 × *g* at 4°C for 20 min. The resultant supernatant was ready for analysis with an HPLC-MS/MS system. HPLC-MS/MS analyses were performed using a UHPLC chromatography system (ExionLC™ AD, SCIEX, America) equipped with a QTRAP^®^ 6500 + mass spectrometer (SCIEX, America) in Novogene Co., Ltd. (Beijing, China). The extracts were injected into a column (Xselect HSS T3, 2.5 μm, 2.1 × 150 mm, Waters) with a 20 min linear gradient at a 0.4 ml/min flow rate for the positive/negative polarity mode. The samples were detected with multiple reaction monitoring (MRM) and based on Novogene’s self-built database (Luo et al., 2015). The Q1 and Q3, retention time, and declustering potential, as well as collision energy, were used for the identification of metabolites. The data files produced by HPLC-MS/MS were processed for peak integration and correction with SCIEX OS software (Version 1.4). The intensities of the peaks were normalized with metaX software. These metabolites were annotated by applying the KEGG database^1^, Lipidmaps database^2^, and HMDB database.^3^

### Data analysis

Analyses of two-way ANOVA were conducted using SPSS 16.0 (SPSS Inc., Chicago, IL, United States) to assess the effect of CO_2_ concentration and sampling time on the physiological traits of *O. alismoides* plants, including acidity, the contents of malate, starch, soluble carbohydrates, soluble proteins, and free amino acids, as well as the activities of Rubisco, PEPC, and PPDK, with CO_2_ concentration and sampling time as factors, and their two-way interactions. Means were significantly different at *p* < 0.05 based on Duncan’s and Tukey’s *post hoc* tests.

The effect of different CO_2_ and sampling times on the metabolic phenotypes of *O. alismoides* were assessed by running principal component analysis (PCA) with metaX. To further discriminate CO_2_ and circadian rhythm response, two pairwise comparisons of the metabolome (LC light vs. HC light, LC dark vs. HC dark) were implemented based on the supervised partial least squares discriminant analysis (PLS-DA). The differentially expressed metabolites were selected with the following screening criteria: (1) high confidence (variable importance in the projection, VIP > 1) in discriminations between LC in the light vs. HC in the light, as well as between LC in the dark vs. HC in the dark; (2) mean intensities in LC-treated plants different from those in HC-treated *O. alismoides* (*p*-value calculated with an independent *t*-test was less than 0.05). For clustering heat maps, the data were normalized using *z*-scores of the intensity areas of differential metabolites and then were produced using the Pheatmap package in R language.

## Results

### Daily fluctuation of acidity, malic acid, and starch

**FIGURE 1:**
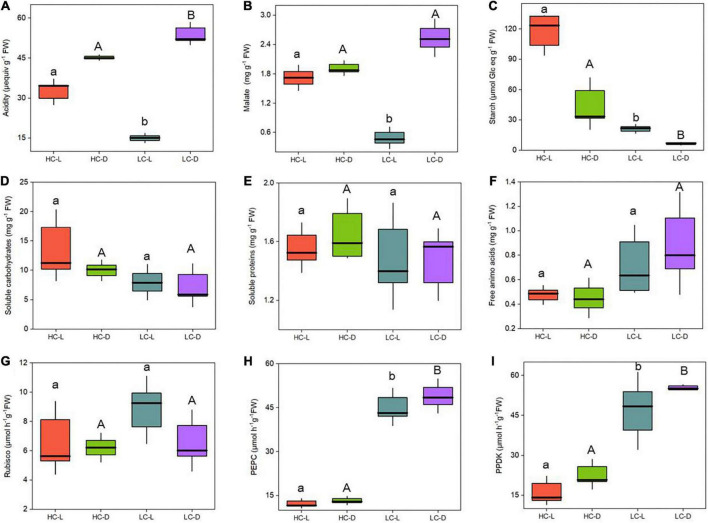
Influence of CO_2_ concentrations on the physiological parameters from *Ottelia alismoides* plants sampled at 21:30 (toward the end of the light period) and 07:30 (toward the end of the dark period). **(A)** Acidity. **(B)** Malate content. **(C)** Starch content. **(D)** Soluble carbohydrates content. **(E)** Soluble protein content. **(F)** Free amino acid content. **(G)** Rubisco activity. **(H)** PEPC activity. **(I)** PPDK activity. The enzyme activities (Rubisco, PEPC, and PPDK) were measured from the rate of disappearance of NADH at 340 nm. HC-L, high CO_2_ in the light; HC-D, high CO_2_ in the dark; LC-L, low CO_2_ in the light; LC-D, low CO_2_ in the dark. Different lowercase letters and uppercase letters indicate significant differences (*p* < 0.05) between different CO_2_ treatments in light and dark, respectively.

Across the high and low CO_2_ conditions, the content of acidity varied between 15 and 33 μequiv g^–1^ FW in the light and 45 and 53 μequiv g^–1^ FW in the dark (Figure 1A). There was a significant difference in diurnal acidity (∼38 μequiv g^–1^ FW) in LC-grown *O. alismoides* (two-way ANOVA, *p* < 0.05; Figure 1A and Table 1); conversely there was no diurnal acidity variation in HC-treated plants (two-way ANOVA, *p* > 0.05; Figure 1A and Table 1). The level of malic acid did not fluctuate between light and dark at HC plants (two-way ANOVA, *p* > 0.05; Figure 1B and Table 1). In contrast, LC-treated plants showed significant light/dark oscillation in malic acid content (two-way ANOVA, *p* < 0.05; Figure 1B and Table 1). Moreover, the concentration of malic acid in the light was only 19% of that in the dark, showing an obvious depletion of malate during the light period at low CO_2_ (Figure 1B). However, the malic acid content in the dark did not change with CO_2_ concentrations (two-way ANOVA, *p* > 0.05; Figure 1B and Table 1). Similar to the change of malic acid, plants under LC showed significant light/dark fluctuation in starch content, which was also present in HC-treated plants (two-way ANOVA, *p* < 0.05; Figure 1C and Table 1). The amount of starch was significantly higher (5.6-times) at HC acclimated *O. alismoides* than that in LC in the light (two-way ANOVA, *p* < 0.05; Figure 1C). Compared to that in the light, the starch content present in the dark was reduced by 70% for LC-grown leaves, while in HC, this reduction was only 63% (Figure 1C).

**TABLE 1:** Two-way ANOVA results for physiological parameters in *Ottelia alismoides*, with CO_2_ concentration and sampling time as factors.

Variables	Source					
	**CO_2_**		**Time**		**CO_2_ × Time**	
	**F**	***P*-value**	**F**	***P*-value**	**F**	***P*-value**

Acidity	0.143	0.770	3.667	0.306	42.571	**0.000**
Malic acid	0.108	0.789	1.430	0.443	36.429	**0.000**
Starch	4.376	0.284	2.143	0.382	9.742	**0.014**
Soluble carbohydrates	2.859	0.129	0.663	0.439	0.150	0.708
Soluble proteins	0.479	0.509	0.122	0.736	0.029	0.868
Free amino acids	3.792	0.087	0.213	0.657	0.435	0.528
Rubisco	1.458	0.440	1.241	0.466	0.924	0.365
PEPC	460.329	**0.030**	2.790	0.343	0.376	0.557
PPDK	2320.000	**0.013**	118.269	0.058	0.020	0.892

Significant *p*-values (*p* < 0.05) are shown in bold. The degrees of freedom = 1 in all cases.

### Soluble carbohydrates, soluble proteins, and free amino acids

Growth in low or high CO_2_ did not have a statistically significant effect on soluble carbohydrates, soluble proteins, and free amino acids of *O. alismoides*, although the soluble carbohydrates were slightly lower and the free amino acids were slightly higher at low vs. high CO_2_ (two-way ANOVA, *p* > 0.05; Figures 1D–F and Table 1).

### Photosynthetic enzymes activity

The Rubisco activity was not statistically significant (two-way ANOVA, *p* > 0.05; Figure 1G and Table 1) between LC and HC treatments in both light and dark. When compared with high CO_2_, the PEPC activity was ∼3 times higher at low CO_2_ in both light and dark (two-way ANOVA, *p* < 0.05; Figure 1H and Table 1). However, the PEPC activity did not differ significantly between light and dark, regardless of the CO_2_ treatments (two-way ANOVA, *p* > 0.05; Figure 1H and Table 1). PPDK displayed a similar variation pattern to PEPC. Compared to HC, LC treatment triggered ∼2 times increase in PPDK activity in *O. alismoides* (two-way ANOVA, *p* < 0.05; Figure 1I and Table 1).

### Overview of the metabolic profiles of all *Ottelia alismoides* samples

**FIGURE 2:**
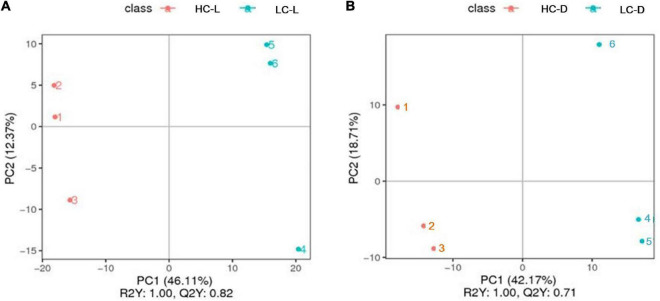
Partial least-squares discriminate analysis (PLS-DA) score plots of *Ottelia alismoides* metabolic profiles for different pairwise comparisons. **(A)** Low CO_2_ in the light vs. high CO_2_ in the light, **(B)** low CO_2_ in the dark vs high CO_2_ in the dark. HC-L, high CO_2_ in the light; HC-D, high CO_2_ in the dark; LC-L, low CO_2_ in the light; LC-D, low CO_2_ in the dark.

To better understand the potential effects of low CO_2_ on *O. alismoides*, as well as comprehensively reveal the carbon and nitrogen metabolic adjustment in response to different levels of CO_2_, LC-MS was performed to identify low-molecular weight metabolites in HC- and LC-acclimated *O. alismoides* plants. We mainly explored the carbon metabolism (photosynthesis and respiration) and nitrogen assimilation pathways with a metabolomic approach. A total of 790 putative metabolites were detected among all *O. alismoides* samples (Supplementary Figure 1), and the PCA analysis with an unsupervised pattern was used to evaluate the overall experimental variation, as well as to examine the differences in metabolite profiles among all the *O. alismoides* samples. The PCA results showed that all samples were distributed into four separate groups (HC dark, HC light, LC dark, and LC light) according to the first two principal components, PC1 and PC2, which represent 31 and 19% of the total variation, respectively (Supplementary Figure 2). This demonstrated that *O. alismoides* samples in HC and LC groups were clearly separated using the first two components. Furthermore, the PLS-DA score plots displayed a distinct separation between HC and LC groups in the light and dark (Figure 2). The R2Y (represents the interpretation rate of the established model to the Y matrices) and Q2Y (represents the predictive power of the model) were at a high level, which further confirmed that each of the supervised models had a good and valid quality. In addition, the varieties of metabolite profiles including abundant metabolites, visualized through a heat map, were remarkably diverse across all samples (Supplementary Figure 3). Taken together, these results propose that *O. alismoides* experienced different metabolic processes for adapting to different CO_2_ conditions.

### Changes of metabolites in *Ottelia alismoides* in response to different CO_2_ in the light and dark

**FIGURE 3:**
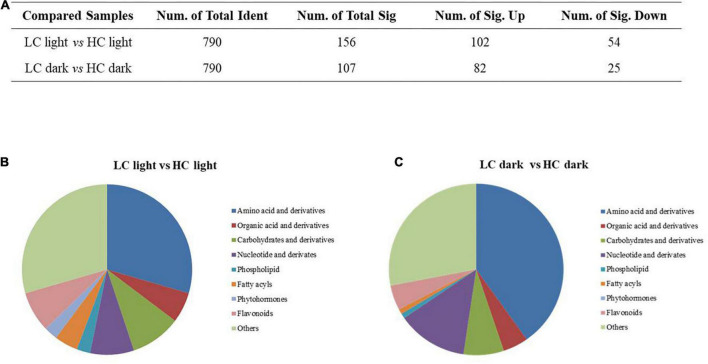
Effects of different CO_2_ on the metabolome of *Ottelia alismoides*. **(A)** Statistics of differently accumulated metabolites in each pairwise comparison. **(B)** Classification of differently accumulated metabolites between low and high CO_2_ treated *O. alismoides* sampled in the light. **(C)** Classification of differently accumulated metabolites between low and high CO_2_ treated *O. alismoides* sampled in the dark.

A *p*-value < 0.05 and a VIP > 1 were utilized to identify the important differently expressed metabolites associated with CO_2_ treatment and circadian rhythm conditions. Compared with high CO_2_, there were 156 differently expressed metabolites at low CO_2_ when in the light, including 102 increased and 54 decreased, whereas when in the dark, there were 107 differently expressed metabolites, including 82 increased and 25 decreased (Figure 3A). The differentially changed metabolites in different CO_2_-treated *O. alismoides* plants mainly included amino acids, organic acids, carbohydrates, nucleotides, phospholipids, and phytohormones (Figures 3B,C).

A total of 55 differentially expressed metabolites were selected, including organic acids, amino acids, and carbohydrates, as shown in Table 2. The corresponding up- or downregulated tendency, indicative of the log_2_ (fold change), showed how these metabolites varied at LC-treated *O. alismoides* plants compared to HC. Among the organic acids, a significant increase was observed in D-galactonic acid (independent *t*-test, *p* < 0.05; Table 2) in light under low CO_2_. As for the amino acids, significantly increased metabolites include glutamate (independent *t*-test, *p* < 0.001 in the light and *p* < 0.05 in the dark; Table 2), glutamine (independent *t*-test, *p* < 0.05 in the light and *p* < 0.01 in the dark; Table 2), γ-aminobutyric acid (GABA) (independent *t*-test, *p* < 0.01 in the light and *p* < 0.05 in the dark; Table 2), lysine (independent *t*-test, *p* < 0.05 in the light and *p* < 0.01 in the dark; Table 2), homoserine, and threonine (independent *t*-test, *p* < 0.05 in both light and dark; Table 2), except the succinic acid, which only increased in light (independent *t*-test, *p* < 0.05; Table 2). On the contrary, tryptophan, serine, and histidine significantly declined under light (independent *t*-test, *p* < 0.05; Table 2). In response to low CO_2_, the upregulated carbohydrates included L-arabinose (3.33-fold in the light, independent *t*-test, *p* < 0.001 and 2.51-fold in the dark, independent *t*-test, *p* < 0.05, respectively; Table 2), 3-phosphoglyceric acid (1.63-fold in the light, independent *t*-test, *p* < 0.05; Table 2), D-xylulose (3.41-fold in the light, independent *t*-test, *p* < 0.05 and 2.81-fold in the dark, independent *t*-test, *p* < 0.05, respectively; Table 2), and D-glucose 1-phosphate (0.94-fold in the light, independent *t*-test, *p* < 0.05 and 0.70-fold in the dark, independent *t*-test, *p* < 0.05, respectively; Table 2). On the contrary, glucose (−0.56-fold in the dark, independent *t*-test, *p* < 0.05; Table 2), fructose (−0.49-fold in the dark, independent *t*-test, *p* < 0.05; Table 2), D-melezitose (−0.97-fold in the light, independent *t*-test, *p* < 0.05; Table 2), and 2-deoxy-D-galactose (−1.18-fold in the light, independent *t*-test, *p* < 0.05 and −0.84-fold in the dark, independent *t*-test, *p* < 0.05, respectively; Table 2) were significantly decreased. Among the phytohormones, 3-indolebutyric acid (IBA) was downregulated considerably (−1.43-fold in the light, independent *t*-test, *p* < 0.05; Table 2) in LC-treated plants in comparison to HC.

**TABLE 2:** List of significantly affected metabolites in low CO_2_ in the light compared to high CO_2_ in the light, and low CO_2_ in the dark compared to high CO_2_ in the dark.

Category of metabolites	log_2_ (LC-L/HC-L)	log_2_ (LC-D/HC-D)
***Carbohydrates and its derivatives***		
D-Glucose 1-phosphate	0.94	0.70
Glucose		−0.56
Fructose		−0.49
L-Arabinose	3.33	2.51
D-Xylose	1.37	1.05
D-Xylulose	3.41	2.81
D-Melezitose	−0.97	
D-Glucose 6-phosphate	0.74	0.99
Glyceraldehyde 3-phosphate		0.48
3-Phosphoglyceric acid	1.63	
2-Deoxy-D-galactose	−1.18	−0.84
D-Xylose	1.37	1.05
D-Ribose	1.59	1.29
D-Sorbitol	−1.42	
***Amino acid and derivatives***		
D-Phenylalanine		0.91
L-Tryptophan	−0.82	
DL- Glutamate	3.21	3.11
L-arginine		1.02
Glutamine	2.09	1.62
Proline	0.79	0.88
Histidine	−0.71	−0.56
γ-Aminobutyric acid	3.94	3.28
Aspartate	1.41	2.27
D-Asparagine	−0.40	
Alanine	1.02	0.58
Lysine	2.17	1.54
Homoserine	1.22	0.77
Threonine	1.22	1.08
DL-Methionine		1.06
DL-Valine		1.11
Leucine	−1.44	1.03
L-Serine	−1.69	
Glycine	1.26	
***Organic acid and its derivatives***		
*cis*-Aconitic acid		
Succinic acid	2.26	
Fumaric acid		
L-Malic acid	−0.87	
D-Galactonic acid	0.77	
Lactic acid		0.81
***Nucleotide and its derivates***		
dADP		
ATP		
CDP		1.61
		
Uridine	1.96	1.48
Adenosine	1.47	
***Fatty acyls***		
Methylenesuccinic acid	2.09	2.83
Octadecadien-6-ynoic acid		
***Phytohormones***		
IBA	−1.43	
***Others***		
Betaine		
Protostemonine	−2.39	
Tropine	−0.90	
Cinnamic acid	−1.20	−0.81
Shikimate	0.79	
2,3-Dihydroxybenzoic acid	1.51	
Idaein chloride		
Peonidin chloride	0.85	

The relative concentration of each metabolite is an average of data from three biological replicates. The numbers represent log_2_ (fold-changes). Red shading means upregulation and green shading means downregulation. LC-L, low CO_2_ in the light; HC-L, high CO_2_ in the light; LC-D, low CO_2_ in the dark; HC-D, high CO_2_ in the dark. 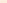
up p<0.05 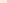
up p<0.01 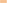
up p<0.001 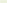
down p<0.05 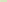
down p<0.01 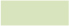
down p<0.001

### Metabolic pathway of differential metabolites in *Ottelia alismoides* under different CO_2_

These above-mentioned metabolites found to be involved with carbon metabolism, assimilation of amino acids, and biosynthesis of secondary metabolites were assorted into different groups based on their metabolic functions, according to the analysis of KEGG pathways, which is the major pathway-related public database that includes genes and metabolites. Based on these metabolomics results, we primarily focused on the key metabolites participating in the vital carbon and nitrogen metabolic pathways in *O. alismoides* plants, such as glycolysis, pentose phosphate pathway (PPP), TCA cycle, and amino acid metabolism.

**FIGURE 4:**
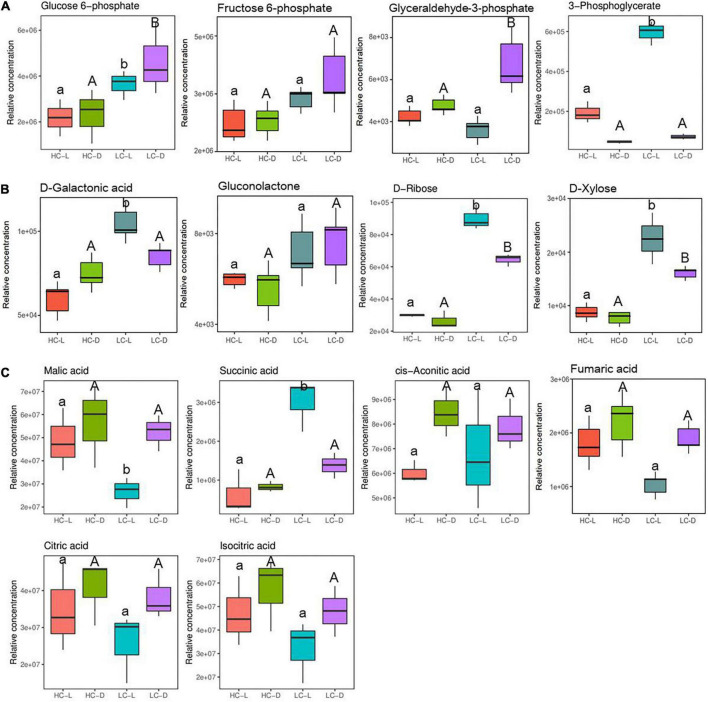
Box plots of relative abundance of the metabolites related to the C metabolism pathway in *Ottelia alismoides*, based on the analysis of KEGG pathways. **(A)** Glycolytic pathway; **(B)** PPP; **(C)** TCA cycle. HC-L, high CO_2_ in the light; HC-D, high CO_2_ in the dark; LC-L, low CO_2_ in the light; LC-D, low CO_2_ in the dark. Different lowercase letters and uppercase letters indicate significant differences (*p* < 0.05) between different CO_2_ treatments in the light and dark, respectively.

Metabolites associated with energy metabolism including glycolysis, PPP, and TCA cycle intermediates were altered significantly following low CO_2_ stress in *O. alismoides* plants (Figure 4). The relative abundance of glucose-6-phosphate (G-6-P, 0.74-fold in the light, independent *t*-test, *p* < 0.05 and 0.99-fold in the dark, independent *t*-test, *p* < 0.05, respectively), glyceraldehyde 3-phosphate (G-3-P, 0.48-fold in the dark, independent *t*-test, *p* < 0.05), and 3-phosphoglyceric acid (3-PG, 1.63-fold in the light, independent *t*-test, *p* < 0.05) significantly increased in LC-treated *O. alismoides* plants (Figure 4A and Table 2). Pentose phosphate pathway, an alternative branch of glycolysis to produce sugars, appeared to be highly activated in *O. alismoides* under low CO_2_ based on the metabolites present in this pathway. The levels of D-ribose (1.59-fold in the light, independent *t*-test, *p* < 0.001 and 1.29-fold in the dark, independent *t*-test, *p* < 0.05, respectively), D-galactonic acid (0.77-fold in the light, independent *t*-test, *p* < 0.05), and D-xylose (1.37-fold in the light, independent *t*-test, *p* < 0.05 and 1.05-fold in the dark, independent *t*-test, *p* < 0.05, respectively), significantly increased in LC-treated *O. alismoides*, respectively (Figure 4B and Table 2). Figure 4C shows the content changes of six organic acids involved in the TCA cycle in light and dark, including citric acid, *cis*-aconitic acid, isocitric acid, succinic acid, fumaric acid, and malic acid in low and high CO_2_ conditions, respectively. Malic acid, the key nocturnal carbon storage metabolite for CAM, cycled as expected with the lowest level in the light and with the highest level in the dark, when under low CO_2_ (independent *t*-test, *p* < 0.05). However, when under HC, the malic acid levels did not differ between light and dark (independent *t*-test, *p* > 0.05). In addition, we observed significantly elevated levels of succinic acid in LC-treated *O. alismoides* in light (2.26-fold, independent *t*-test, *p* < 0.05; Figure 4C and Table 2). The other metabolites in the TCA cycle (citric acid, *cis*-aconitic acid, fumaric acid, and isocitric acid) have no significant alterations under LC when compared to HC (independent *t*-test, *p* > 0.05; Figure 4C and Table 2).

**FIGURE 5:**
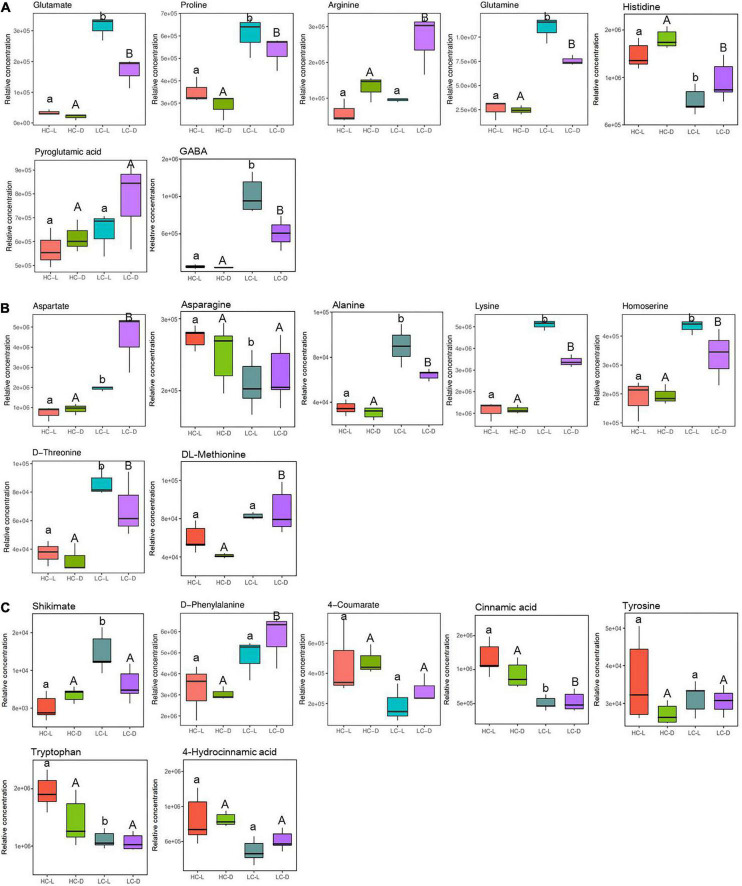
Box plots of relative abundance of the metabolites related to N metabolism pathway in *Ottelia alismoides*, based on the analysis of KEGG pathways. **(A)** Arginine and proline metabolism; **(B)** aspartate metabolism; **(C)** shikimate–phenylpropanoid metabolism. HC-L, high CO_2_ in the light; HC-D, high CO_2_ in the dark; LC-L, low CO_2_ in the light; LC-D, low CO_2_ in the dark. Different lowercase letters and uppercase letters indicate significant differences (*p* < 0.05) between different CO_2_ treatments in the light and dark, respectively.

Figure 5A shows the content changes of glutamate and its derivatives including arginine, proline, glutamine, and GABA in light and dark. Glutamate was observed to increase (3.21-fold in the light, independent *t*-test, *p* < 0.001 and 3.11-fold in the dark, independent *t*-test, *p* < 0.05, respectively) in LC-treated *O. alismoides* plants (Figure 5A and Table 2). Consistent with the glutamate increases, arginine (1.02-fold in the dark, independent *t*-test, *p* < 0.05), proline (0.79-fold in the light, independent *t*-test, *p* < 0.05 and 0.88-fold in the dark, independent *t*-test, *p* < 0.05, respectively), glutamine (2.09-fold in the light, independent *t*-test, *p* < 0.05 and 1.62-fold in the dark, independent *t*-test, *p* < 0.01, respectively), and GABA (3.94-fold in the light, independent *t*-test, *p* < 0.01 and 3.28-fold in the dark, independent *t*-test, *p* < 0.05, respectively) also increased in LC-treated *O. alismoides* plants. Contrary, the abundance of histidine (−0.71-fold in the light, independent *t*-test, *p* < 0.05 and −0.56-fold in the dark, independent *t*-test, *p* < 0.05, respectively) decreased under LC (Figure 5A and Table 2). The amino-acid pathway derived from aspartate is in charge of the distribution of carbon flux from aspartate into the synthesis of lysine, threonine, and methionine (Curien et al., 2009). Low CO_2_ caused a significant increase in metabolites produced by aspartate metabolism, including aspartate (1.41-fold in the light, independent *t*-test, *p* < 0.05 and 2.27-fold in the dark, independent *t*-test, *p* < 0.05, respectively), alanine (1.02-fold in the light, independent *t*-test, *p* < 0.05 and 0.58-fold in the dark, independent *t*-test, *p* < 0.05, respectively), homoserine (1.22-fold in the light, independent *t*-test, *p* < 0.05 and 0.77-fold in the dark, independent *t*-test, *p* < 0.05, respectively), threonine (1.22-fold in the light, independent *t*-test, *p* < 0.05 and 1.08-fold in the dark, independent *t*-test, *p* < 0.05, respectively), lysine (2.17-fold in the light, independent *t*-test, *p* < 0.05 and 1.54-fold in the dark, independent *t*-test, *p* < 0.01, respectively), and DL-methionine (1.06-fold in the dark, independent *t*-test, *p* < 0.05) in *O. alismoides* (Figure 5B and Table 2). However, the asparagine content (−0.40-fold in the light, independent *t*-test, *p* < 0.05) decreased in low-CO_2_-treated *O. alismoides* (Figure 5B and Table 2). The shikimate pathway provides carbon sources for the synthesis of aromatic amino acids, such as tryptophan, phenylalanine, and tyrosine; approximately 30% of the fixed carbon is fluxed into this pathway in plants (Maeda and Dudareva, 2012). In the present study, the content of shikimate (0.79-fold in the light, independent *t*-test, *p* < 0.05) and phenylalanine (0.91-fold in the dark, independent *t*-test, *p* < 0.05) were significantly increased under LC treatment. In contrast, tryptophan was significantly decreased (−0.82-fold in the light, independent *t*-test, *p* < 0.05) under LC. In addition, the metabolites that were derived from phenylalanine in the shikimate pathway, cinnamic acid (−1.20-fold in the light, independent *t*-test, *p* < 0.05 and −0.81-fold in the dark, independent *t*-test, *p* < 0.05, respectively) also significantly decreased in abundance under LC (Figure 5C and Table 2). No significant difference was observed in tyrosine and 4-hydrocinnamic acid levels (independent *t*-test, *p* > 0.05; Figure 5C).

## Discussion

The synergistic regulation of carbon and nitrogen metabolism plays a crucial role in the adaptation of plants to all kinds of stresses. For freshwater plants, the availability of CO_2_ is often one of the growth-limiting factors. To understand how the freshwater plant *O. alismoides* regulates carbon and nitrogen metabolism for better adapting to environmental low CO_2_ limitations, physiological measurements combined with a comprehensive metabolomics analysis were performed in *O. alismoides* acclimated to high and low CO_2_, respectively. In this section, the physiological and biochemical relevance of the present data, and the differential roles of metabolites involved in CAM metabolism, respiratory metabolism, and amino acid metabolism were discussed to reveal how the carbon and nitrogen metabolism were co-regulated when *O. alismoides* acclimated to low CO_2_. To the best of our knowledge, this is the first study to reveal the metabolic response of C and N metabolism to low CO_2_ stress in the freshwater plant *O. alismoides* with metabolome.

### Induced CAM metabolism in *Ottelia alismoides* in response to low CO_2_

In aquatic ecosystems, less than 10% of the tested aquatic species have been discovered to operate CAM photosynthesis, which has been considered to be a carbon-conservation strategy that effectively increases carbon utilization (Maberly and Gontero, 2017). The features of CAM generally include the following: (1) dark fixation of CO_2_ and nocturnal accumulation of malic acid, (2) diurnal fluctuations in acidity/malate and starch content, and the opposite diel pattern of acidity/malate and starch, (3) high activity of PEPC at night that allows the production of malate, and (4) high ratio of PEPC:rubisco activity (Keeley, 1998; Igamberdiev and Eprintsev, 2016; Maberly and Gontero, 2017). In the present study, only low-CO_2_-acclimated *O. alismoides* exhibited significant diurnal titratable acidity and malate fluctuations, and an opposite diel pattern of starch change, consistent with the expectation and characteristics of CAM metabolism. Furthermore, based on the enzymatic activity analysis (Rubisco, PEPC, and PPDK), when *O. alismoides* were treated with low CO_2_, the activities of PEPC and PPDK (regenerates PEP to ensure the substrate requirements for PEPC) were very high in the dark and were much higher than that of HC plants, in accordance with nocturnal carboxylation due to CAM photosynthesis. The increased enzyme activity of PEPC and PPDK induced by low CO_2_ was consistent with the previous reports for *O. alismoides* (Zhang et al., 2014; Shao et al., 2017) and was consistent with that in low-CO_2_-grown terrestrial CAM species *Opuntia ficus-indica* (Israel and Nobel, 1994) and the very low-CO_2_-treated microalgae *Nannochloropsis oceanica* (Wei et al., 2019). Overall, these patterns of variation of enzyme activities, as well as acidity and starch content, confirm that CAM photosynthesis was induced in LC-stressed *O. alismoides*, which confirmed our previous results (Huang et al., 2018; Han et al., 2020).

### Increased respiration including glycolysis, pentose phosphate pathway, and tricarboxylic acid cycle in *Ottelia alismoides* in response to low CO_2_

**FIGURE 6:**
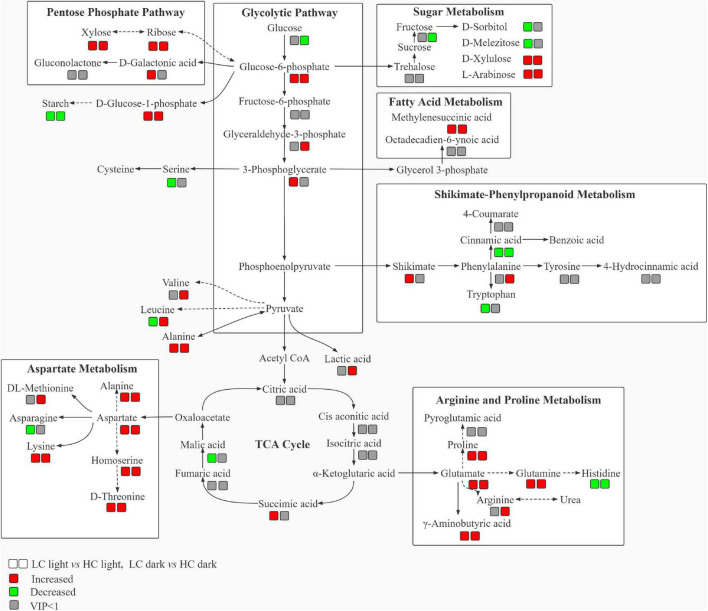
Schematic diagram of the proposed metabolic network in *Ottelia alismoides* responding to low and high CO_2_. Results represent fluctuations performing pairwise comparisons; the symbols underneath the metabolite names correspond to the result of the pairwise comparison that is indicated in the legend. Dashed lines symbolize multi-step or not fully elucidated pathway sections and solid lines symbolize one-step consecutive metabolites in a biosynthetic pathway.

**FIGURE 7:**
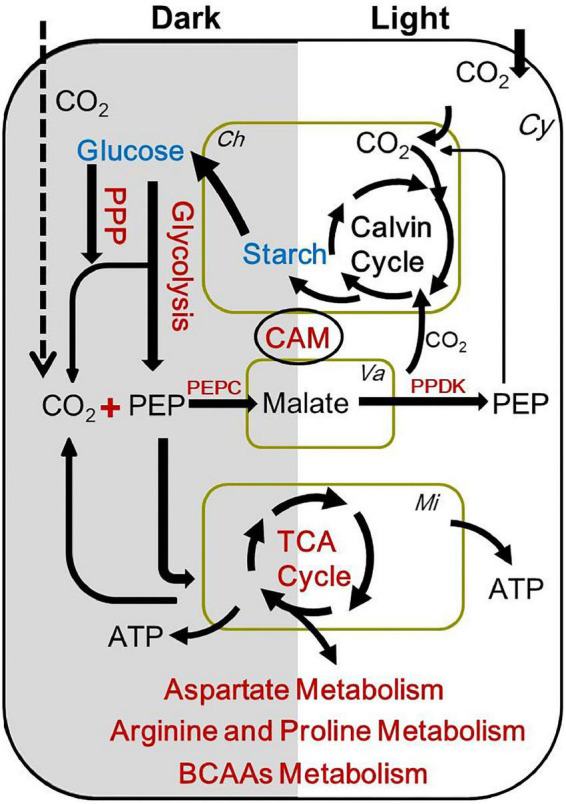
A summary of the metabolic changes in carbon and nitrogen metabolism in low CO_2_ compared to high CO_2_ grown *Ottelia alismoides*. Red indicates an increased metabolite content/upregulated reaction/induced metabolism and blue indicates a decreased metabolite content as compared with high CO_2_ grown plants. The dotted line indicates that the CO_2_ in solution perhaps enters the plant cell in dark for CAM carbon fixation. Ch, chloroplast; Cy, cytoplasm; Mi, mitochondria; Va, vacuole; PEP, phosphoenolpyruvate; BCAAs, branched-chain amino acids; PPP, pentose phosphate pathway.

It has been pointed out that an active CAM would suffer energy costs for investing in the machinery and running of CAM (Raven and Lucas, 1985). Glycolysis, PPP, and TCA cycle are primary metabolic pathways in plants and could provide energy and carbon skeletons for other metabolic pathways. According to the metabolomic analyses, a metabolic network (Figure 6) and a summary of metabolic changes in carbon and nitrogen metabolism (Figure 7) were reconstructed for *O. alismoides* under different CO_2_ conditions. Glucose-6-phosphate is linked to the start of both glycolysis and PPP pathways, and these are critical for the plants to respond to abiotic stress (Aranda et al, 2020). Glycolysis is the predominant pathway to fuel plant respiration; in addition, it is directly involved in many biochemical adaptations of plants to environmental stresses, such as nutrient limitation, as well as osmotic, drought, and cold stresses, which are crucial in plants (Plaxton, 1996). In the present study, the upregulation of the metabolites (G-6-P, G-3-P, and 3-PG) involved in glycolysis under LC indicates that there was a higher accumulation of intermediates of the glycolytic pathway, which presumably reflects some adjustment in the pattern of carbon flux responding to reduced photosynthesis and higher energy demand in low-CO_2_-acclimated *O. alismoides*. In addition, the changed carbohydrates were not only involved in glycolysis but also related to PPP, as revealed in this study. In detail, increased levels of D-ribose, D-xylose, and D-galactonic acid derived from the PPP pathway were observed in LC-treated plants, reflecting increased PPP metabolism in these plants. Correspondingly, the general decrease of glucose and fructose under low CO_2_ was most likely caused by increased glycolysis and PPP pathways (Hasler-Sheetal et al., 2016). The biological pathway analysis further confirms that the PPP pathway was altered by LC treatment (Supplementary Figure 4). A similar promotion of glycolysis and PPP has also been observed previously in other species, such as wheat cultivars under drought stress (Bowne et al., 2012). Pyruvate, the final metabolite of glycolysis, failed to be detected through metabolomic analysis in the present study, however, the amino acids derived from pyruvate, alanine, valine, and leucine were significantly upregulated in LC-treated *O. alismoides*. These results imply that when *O. alismoides* plants were treated with LC, they increased glycolytic flux and consumed carbohydrate pools to meet the requirements of energy and intermediate substance for the biosynthesis of other metabolites. As the core of respiratory machinery, the improved activity of the TCA cycle indicates the elevated energy demand and metabolic rate (Zhang et al., 2021). As revealed by the metabolomic data, six metabolites were identified in the TCA cycle in the present study. The succinic acid was found to be significantly increased in LC-treated *O. alismoides* plants. This finding is consistent with the result from Hasler-Sheetal et al. (2015), who reported that succinic acid was significantly increased in anoxic-stressed seagrass *Zostera marina*. The elevation of TCA pathway intermediates probably indicates the activation of the TCA cycle in LC-treated *O. alismoides*, which was in accordance with the report of Tcherkez et al. (2008), who clarified that the TCA cycle activity and glutamate production are promoted under a low CO_2_ environment. Wei et al. (2019) also recently reported that the TCA cycle genes were enriched in low-CO_2_-grown microalgae *Nannochloropsis oceanica* by tracking the transcriptomic profiles.

Taken together, these observations indicate that the respiratory metabolism was upregulated in LC-treated *O. alismoides*, which was consistent with the previous study (Tcherkez et al., 2008). As we mentioned above, CAM photosynthesis was induced in *O. alismoides* under low CO_2_. Aquatic CAM photosynthesis is a CO_2_ concentrating mechanism that evolved in response to carbon limitation in water (Keeley, 1998). During the operation of CAM, the aquatic CAM plants are capable of dark fixation of CO_2_ and storage of malic acid, and daytime decarboxylation of malic acid to release CO_2_, which reduces carbon limitation, but with a certain investment of energy. It is therefore likely that when *O. alismoides* was stressed by LC, CAM photosynthesis was induced to adapt to the carbon stress, and the respiration was correspondingly upregulated to provide more energy for sustaining CAM photosynthesis.

### Increased amino acid metabolism in *Ottelia alismoides* in response to low CO_2_

Nitrogen metabolism regulation, which is closely linked with carbon metabolism in basic biochemical pathways in plants, is crucial for plant stress tolerance (Lawlor, 2002; Zhong et al., 2017). The carbon skeletons for amino acid synthetic pathways are generated in different sectors of the respiration process. Furthermore, ATP and reductant required for amino acid biosynthesis are also from respiration. Thus, nitrogen assimilation inevitably closely interacts with respiration, and the C produced by photosynthetic CO_2_ assimilation is a building block for amino acid synthesis (Stitt et al., 2002). In the present study, low CO_2_ treatment significantly affected the nitrogen metabolism and had a great impact on the amino acids pool in *O. alismoides*. Most of the identified amino acids, as well as a number of N-containing molecules derived from glutamine and glutamate, such as GABA, were strongly induced under low CO_2_ stress. These differentially changed amino acids were mainly involved in pathways of glutamate and arginine metabolism, aspartate metabolism, and branched-chain amino acids (BCAAs) metabolism.

The amino acids involved in glutamate and arginine metabolism, including glutamate, glutamine, proline, GABA, and arginine (only in the dark), were derived from TCA cycle intermediate α-ketoglutaric acid and were all significantly promoted under LC. Both glutamate and glutamine have an important effect on nitrogen metabolism (Zhao et al., 2018). The increase in glutamate and glutamine content under LC was in line with a previous report in *Arabidopsis* that glutamine level was upregulated when subjected to combined stress with drought and heat (Rizhsky et al., 2004). It is also reported that low N induced significant upregulation of amino acids in plants, including glutamate and glutamate-derived amino acids (Kusano et al., 2011). The gene *glutamate dehydrogenase 2* (*GDH2*), which was involved in the synthesis of glutamate, was upregulated by drought stress in wild species of tomato (Egea et al., 2018). The amino acid GABA is mainly synthesized from glutamate through the catalyzation of glutamate decarboxylase (Michaeli et al., 2011). This metabolite always accumulates rapidly under adverse environmental conditions; moreover, genes involved in the GABA metabolism, *glutamate decarboxylase 1* (*GAD1*) and γ*-aminobutyrate transaminase* (*GABA-T*), were specifically induced by drought stress in the wild relative of tomato, *Solanum pennellii* (Egea et al., 2018). The high level of GABA is tightly correlated with the enhanced resistance to stressful conditions (Kinnersley and Turano, 2000; Renault et al., 2010; Podlešáková et al., 2018). Numerous metabolic processes are regulated by GABA, such as the TCA cycle, nitrogen metabolism, as well as metabolic responses to oxidative stress (Bouché and Fromm, 2004). It has been suggested that seagrass could compensate for the deprivation of energy by utilizing the carbon derived from glutamine *via* the GABA shunt (Hasler-Sheetal et al., 2015). In this study, the significantly increased GABA throughout the diel cycle in low CO_2_ treated plants is in agreement with the previous studies within the low-light-stressed seagrass *Zostera marina* (Hasler-Sheetal et al., 2016). The enhanced GABA perhaps implies a GABA shunt compensated for energy deprivation caused by low carbon limitation in *O. alismoides*.

Concerning other reactions of N metabolism, low CO_2_ also promoted the aspartate-family pathway in *O. alismoides*, revealed by the significantly increased concentration of aspartate, lysine, methionine, threonine, alanine, and homoserine. Lysine, methionine, and threonine, as the essential amino acids, are commonly referred to as aspartate (Asp)-family amino acids; they are generated from a common precursor Asp *via* complicated pathways (Kirma et al., 2012). These Asp-family amino acids originated from TCA intermediates oxaloacetate and contribute to nitrogen storage and utilization. Plenty of evidence has revealed that the Asp-family metabolism is linked with the TCA cycle, which plays an important role in regulating the physiological response to energy deprivation caused by various abiotic stresses in plants (Kirma et al., 2012). Taking lysine as an example, it not only acted as an essential constituent for proteins but also catabolized in the TCA cycle for generating energy (Galili, 2011). The promotion in the Asp-family pathway observed in LC stressed *O. alismoides* suggests that the metabolic response caused by carbon limitation is similar to that found for many other abiotic stresses. In the metabolomic study of a model plant *Lemna minor* L., the amino acids of the Asp families were also upregulated in herbicides in glyphosate-treated plants (Kostopoulou et al., 2020). Interestingly, in this study, asparagine was found to be significantly decreased under low CO_2_ in light. Generally, when under C limitation, N is channeled to asparagine as a transient storage pool through the transcriptionally activated asparagine synthetase (Weigelt et al., 2008). However, the reason for asparagine reduction in this study is unknown.

In addition, alanine, valine, and leucine, which are associated with pyruvate metabolism, were observed to be increased in low-CO_2_-treated *O. alismoides*. Alanine is the major free amino acid and the latter two (valine and leucine) belong to the BCAAs (Pernollet et al., 1986), which are jointly regulated in their biosynthesis due to sharing common enzymes (Joshi et al., 2010). It has been proposed that BCAAs could provide alternative carbon sources for stressed plants. Previous studies have reported that BCAAs’ content was significantly increased in sugar-starved or drought-stressed *Arabidopsis* (Rizhsky et al., 2004; Taylor et al., 2004). Urano et al. (2009) also found the increased accumulation of BCAAs under dehydration stress and BCAAs were transcriptionally regulated. Egea et al. (2018) reported that the gene involved in BCAAs biosynthesis, the *branched-chain-amino-acid transaminase 5* (*BCAT5*), was induced in drought-stressed *Solanum pennellii*. As such, the increased accumulation of BCAAs in low-CO_2_-stressed *O. alismoides* might be associated with the adaptation of plants to carbon deficiency to fulfill the demands of growing in C-limited conditions.

Collectively, these observations indicate that the changes in amino acids caused by low CO_2_ are closely associated with the regulation of intermediates involved in glycolysis, PPP, and TCA cycle. Low CO_2_ promoted the biosynthesis of amino acids in *O. alismoides* through the upregulation of respiration. Wei et al. (2019) have also reported that in low-CO_2_ grown *Nannochloropsis oceanica*, the carbon flow was switched from protein synthesis to other pathways (e.g., gluconeogenesis and secondary metabolite biosynthesis). The enhanced respiration process under low CO_2_ could produce more energy and precursors (such as α-ketoglutaric acid and oxaloacetate) for amino acid synthesis. Moreover, the operation of CAM induced by low CO_2_ is inevitable to increase the demand for energy and other resources such as proteins (enzymes) (Raven and Lucas, 1985; Maberly and Gontero, 2017). Therefore, the promoted N metabolism in low CO_2_, including glutamate and arginine metabolism, aspartate metabolism, and BCAAs metabolism, and these in turn presumably, could be used to support extra energy and alternative carbon sources, as well as to provide components for the construction of CAM machines for better adaptation of *O. alismoides* to carbon limitation conditions.

## Conclusion

In conclusion, this work provides a valuable insight into the metabolic response of *O. alismoides* to variable CO_2_ based on a conjoint analysis of physiology and metabolomics. Comparative metabolomic analysis revealed significant changes in the metabolite profiles, especially the regulation of C and N metabolism, responding to the variable CO_2_ in *O. alismoides*. Nevertheless, more investigation is required to understand the relevance and contributions of these metabolic processes mediated by variable CO_2_.

## Data availability statement

The raw data supporting the conclusions of this article will be made available by the authors, without undue reservation.

## Author contributions

WL and WH designed the experiments. SH and WH performed the experiments and collected the data. WH, SH, and LW analyzed the data. WH prepared the manuscript. All authors contributed and approved the final manuscript.

## Footnotes

1
http://www.genome.jp/kegg/

2
http://www.lipidmaps.org/

3
http://www.hmdb.ca/
