# Supplementary material for: Carbon and nitrogen metabolic regulation in freshwater plant Ottelia alismoides in response to carbon limitation: A metabolite perspective
Source: Front Plant Sci. 2022 Sep 15;13:962622. doi: 10.3389/fpls.2022.962622 (PMC9522611; doi:10.3389/fpls.2022.962622)
Supplement: Supplementary file 1 [file Data_Sheet_1.docx]

**Supplementary Information**

Supplementary Table S1. Conditions in the low and high CO_2_ treatments.

| Conditions | High CO_2_ | Low CO_2_ |
| --- | --- | --- |
| Temperature (°C) | 25 (22-27) | 25 (22-27) |
| pH^a^ | 6.8 (6.7-7.0) | 9.2 (8.1-10) |
| Alkalinity (mequiv L^−1^) | 2.5 (2.0-2.9) | 0.91 (0.46-1.71) |
| CO_2_ (μmol L^−1^) | 649 (457-864) | 1.5 (0.03-11.5) |
| HCO_3_^−^ (mmol L^−1^) | 2.5 (2.0-2.9) | 0.66 (0.16-1.6) |

*Mean values are given with ranges in parentheses.*

*^a^ calculated as a geometric mean.*


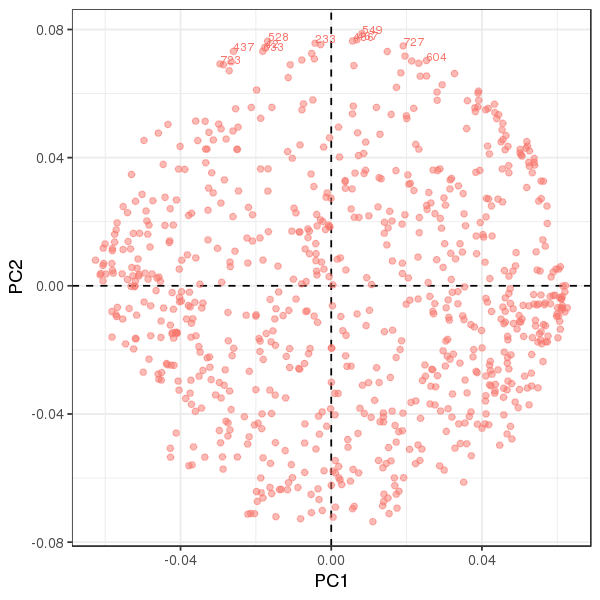


Supplementary Figure S1. The PCA loading plots of the identified metabolites in *Ottelia alismoides* treated with different CO_2_.


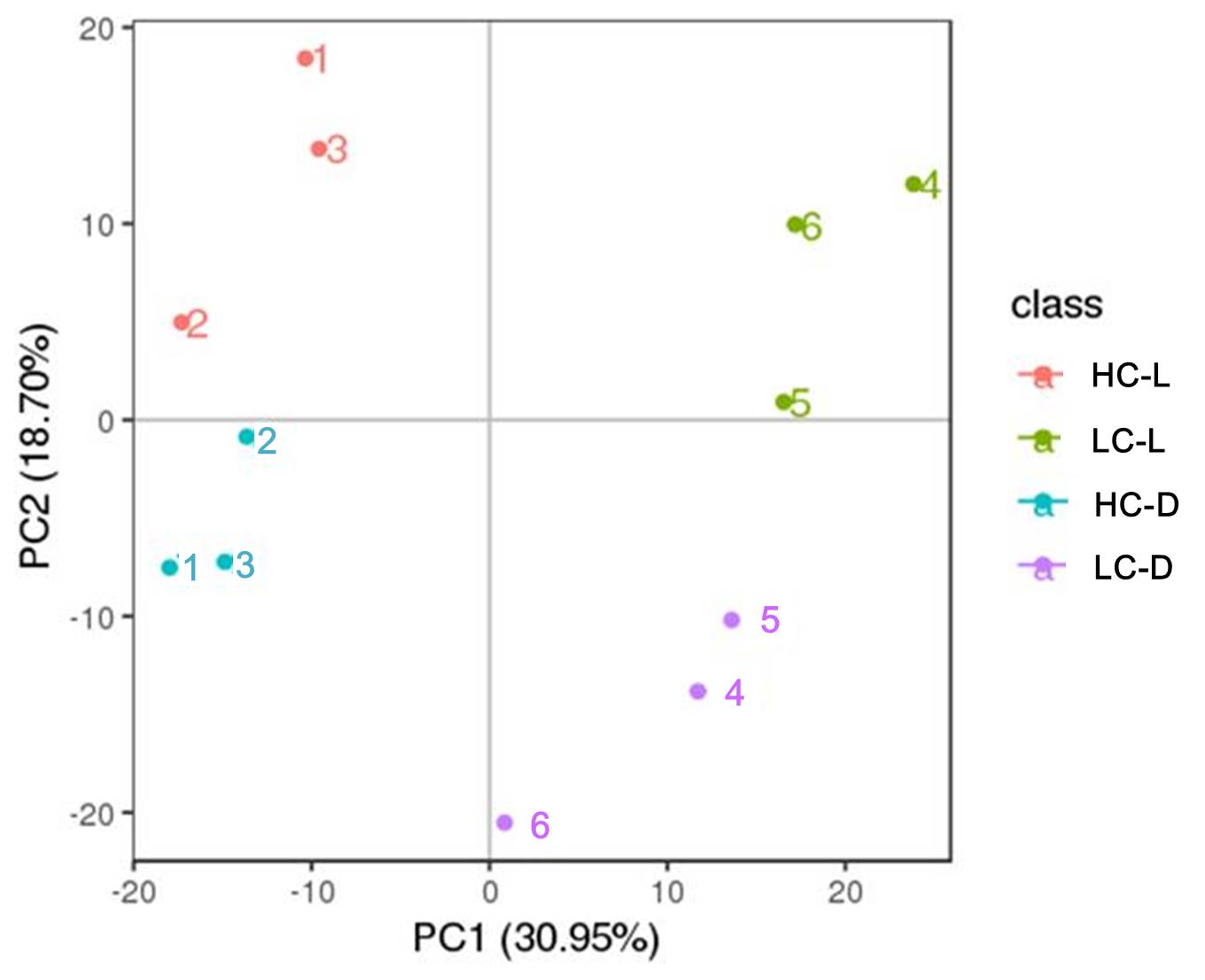


Supplementary Figure S2. The PCA score plots of metabolic profiles in *Ottelia alismoides* treated with high and low CO_2_. HC-L, high CO_2_ in the light; LC-L, low CO_2_ in the light; HC-D, high CO_2_ in the dark; LC-D, low CO_2_ in the dark. PCA represents a total of 49.65% variation, out of which 30.95% of the variation is represented in PC1, and 18.70% of the variation is represented in PC2.


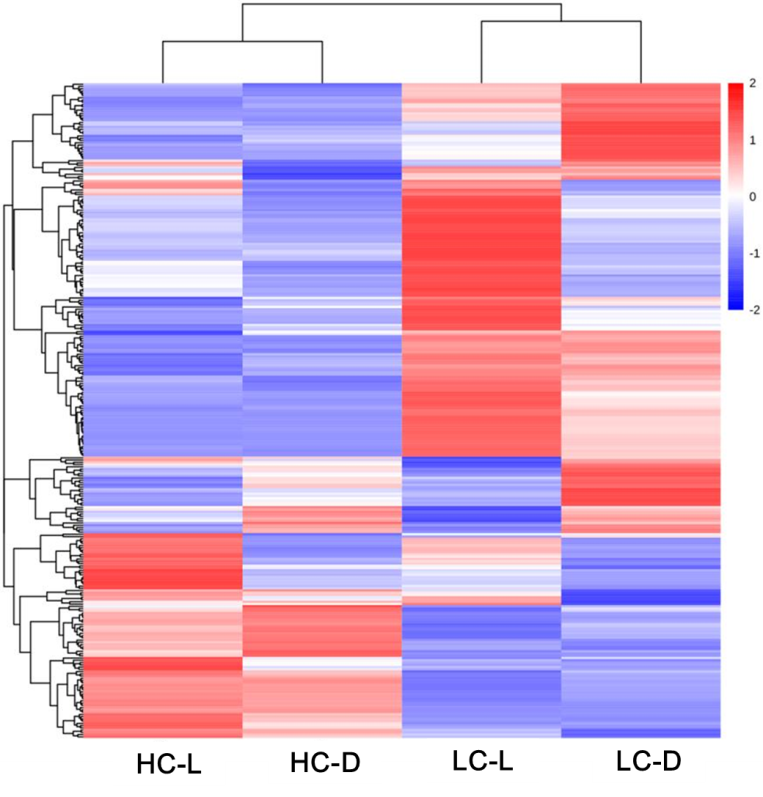


Supplementary Figure S3. Two-dimensional cluster heat map of the recorded GC/MS metabolite profiles of *Ottelia alimoides* following treatments with low and high CO_2_ sampled in the light and dark. The rows correspond to metabolites and the columns correspond to the treatments being performed. Each cell is color-coded based on the relative concentration of the corresponding metabolite using a color-scale ranging from -2 (light blue), indicating low values, to 2 (light red) indicating high values. HC-L, high CO_2_ in the light; HC-D, high CO_2_ in the dark; LC-L, low CO_2_ in the light; LC-D, low CO_2_ in the dark.


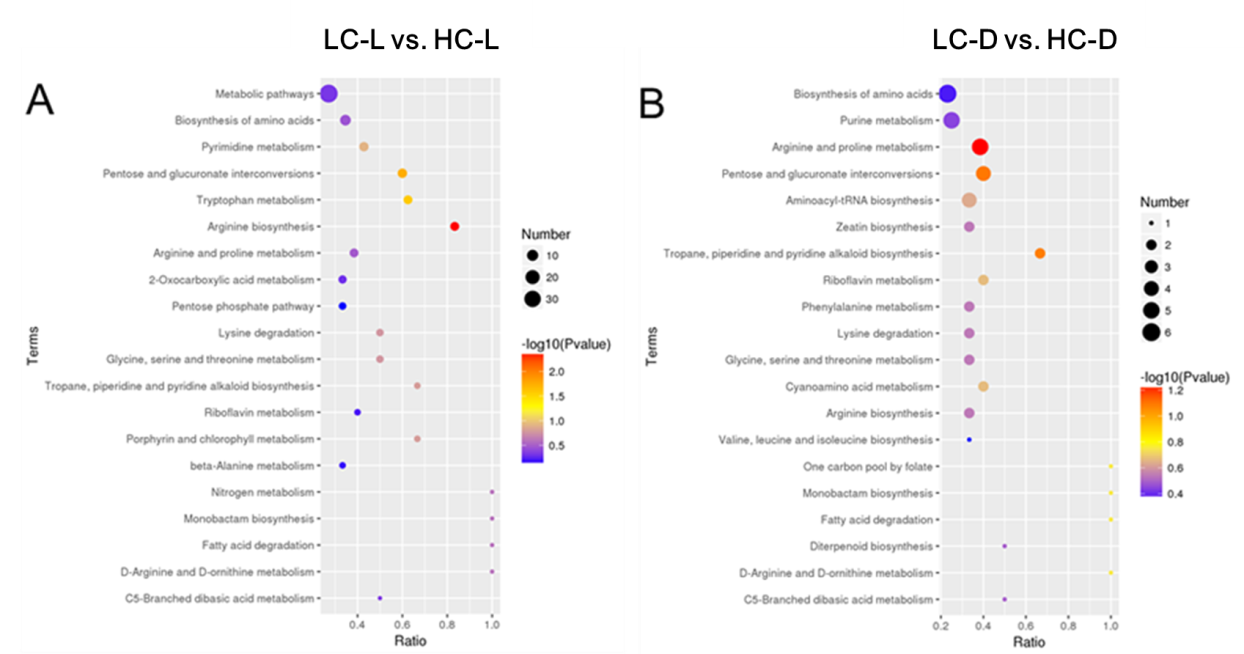


Supplementary Figure S4. Pathway enrichment analysis showing significantly altered metabolic pathways in *Ottelia alimoides* in response to different CO_2_. (A) low CO_2_ in the light vs high CO_2_ in the light, (B) low CO_2_ in the dark vs high CO_2_ in the dark. The circle color represents p-value, the smaller the p-value is, the more reliable the enrichment significance is. The size of circle indicates the number of differentially expressed metabolites enriched in the pathway, and the larger the circle, the more abundant the metabolites is. LC-L, low CO_2_ in the light; HC-L, high CO_2_ in the light; LC-D, low CO_2_ in the dark; HC-D, high CO_2_ in the dark.
